# Affinin (Spilanthol), Isolated from *Heliopsis longipes*, Induces Vasodilation via Activation of Gasotransmitters and Prostacyclin Signaling Pathways

**DOI:** 10.3390/ijms18010218

**Published:** 2017-01-22

**Authors:** Jesús Eduardo Castro-Ruiz, Alejandra Rojas-Molina, Francisco J. Luna-Vázquez, Fausto Rivero-Cruz, Teresa García-Gasca, César Ibarra-Alvarado

**Affiliations:** 1 Laboratorio de Biología Celular y Molecular, Facultad de Ciencias Naturales, Universidad Autónoma de Querétaro, Campus Juriquilla, 76230 Querétaro, Qro., Mexico; dentaqro@live.com.mx; 2 Laboratorio de Investigación Química y Farmacológica de Productos Naturales, Facultad de Ciencias Químicas, Universidad Autónoma de Querétaro, Centro Universitario, 76010 Querétaro, Qro., Mexico; rojasa@uaq.mx (A.R.-M.); fjlunavz@yahoo.com.mx (F.J.L.-V.); 3 Departamento de Farmacia, Facultad de Química, Universidad Nacional Autónoma de México, Ciudad Universitaria, 04510 México, D.F., Mexico; joserc@unam.mx

**Keywords:** *Heliopsis longipes*, affinin, vasodilation, rat aorta, gasotransmitters, prostacyclin

## Abstract

*Heliopsis longipes* roots have been widely used in Mexican traditional medicine to relieve pain, mainly, toothaches. Previous studies have shown that affinin, the major alkamide of these roots, induces potent antinociceptive and anti-inflammatory activities. However, the effect of *H. longipes* root extracts and affinin on the cardiovascular system have not been investigated so far. In the present study, we demonstrated that the dichloromethane and ethanolic extracts of *H. longipes* roots, and affinin, isolated from these roots, produce a concentration-dependent vasodilation of rat aorta. Affinin-induced vasorelaxation was partly dependent on the presence of endothelium and was significantly blocked in the presence of inhibitors of NO, H_2_S, and CO synthesis (N^G^-nitro-l-arginine methyl ester (l-NAME), dl-propargylglycine (PAG), and chromium mesoporphyrin (CrMP), respectively); K^+^ channel blockers (glibenclamide (Gli) and tetraethyl ammonium (TEA)), and guanylate cyclase and cyclooxygenase inhibitors (1*H*-[1,2,4]oxadiazolo[4,3-*a*]quinoxalin-1-one (ODQ) and indomethacin (INDO), respectively). Our results demonstrate, for the first time, that affinin induces vasodilation by mechanisms that involve gasotransmitters, and prostacyclin signaling pathways. These findings indicate that this natural alkamide has therapeutic potential in the treatment of cardiovascular diseases.

## 1. Introduction

*Heliopsis longipes* (A. Gray) S. F. Blake (Asteraceae) (*H. longipes*) is an herbaceous plant native to Mexico, that grows particularly in the states of Querétaro, Guanajuato, and San Luis Potosí, where it is known by common names including “Chilcuague”, “Chilcuán”, “Chilmecatl”, “Aztec root”, “Golden root”, among others [1,2,3,4]. In Central Mexico, the roots of this species are widely used as a spice, home insecticide, and for the treatment of some illnesses, which include toothaches, gingival disease, and muscular pain [5,6,7,8]. When *H. longipes* roots come into contact with oral cavity tissues, they produce numbness and a tingling sensation of the tongue, associated with a significant increase in salivary flow [9,10]. The predominant bioactive molecules found in *H. longipes* roots are *N*-alkylamides or alkamides, mainly *N*-isobutyl-2*E*,6*Z*,8*E*-decatrienamide, also known as affinin or spilanthol [7,11,12,13,14,15,16]. This alkamide is not only found in *H. longipes* roots, it has also been identified in other plants, including *Spilanthes* species (Synonym: *Acmella* species) [17,18,19,20,21,22,23,24]. A variety of biological activities such as larvicidal (10–14 µg/mL) [25], antimicrobial (25–300 µg/mL) [4], fungistatic, and bacteriostatic (5–150 µg/mL) [8] effects have been attributed to this compound. In addition, several pharmacological studies have demonstrated that affinin displays analgesic (ED_50_ = 1 mg/kg intraperitoneal (i.p.) in mice) [5,16], antinociceptive (ED_50_ = 6.98 mg/kg *per os* (p.o.); ED_50_ = 36 ± 5 mg/kg i.p. in mice) [6,26], anti-inflammatory (90–180 µM in macrophage cell line) [18], anxiolytic (3–30 mg/kg i.p. in mice) [6], and diuretic (800 mg/kg p.o. in mice) [27] properties. Some of these pharmacological activities have been also reported for crude organic extracts of *H. longipes* roots [5,6,26,28,29,30,31].

Affinin has an adequate lipophilicity. An in vitro permeability test showed that this alkamide (10 µg/mL) permeates through CaCo-2 cell monolayer cultures via passive diffusion. Whereas in vivo assays demonstrated that it is able to permeate skin and oral mucosa, and subsequently reach blood circulation, and cross the blood-brain barrier in high amounts (~98%) [23,32]. Therefore, this compound might be considered a valuable potential drug candidate [13,18,23,33].

With respect to safety assessment studies, the acute toxicity of affinin was evaluated on ICR mice and the determined median lethal dose (LD_50_ = 113 mg/kg) was significantly higher than the doses required to elicit antinociception [6,26]. No mutagenic effects were observed by using the Ames test [6] and antimutagenic effects of affinin were observed at 25 and 50 µg/mL [10]. The cytotoxic effect of affinin was determined on human HEK293 kidney cells and the calculated mean inhibitory concentration (IC_50_) was 260 µg/mL, while the concentration used to observe biological effects was 100 µg/mL [27]. No cytotoxic effects of affinin, which elicits a stimulatory effect on nitric oxide (NO) production in RAW 264.7 murine macrophages, were observed at concentrations up to 40 µg/mL [18].

Regarding the mechanism of action underlying the antinociceptive effect of affinin, Déciga-Campos et al. [26] showed that this effect might be due to activation of opiodergic, serotoninergic, and GABAergic systems, and also involves participation of the NO/cGMP/potassium channel pathway. It has been well documented that this signaling pathway plays an important role in vascular tone regulation [34,35,36,37,38,39]. This physiological process is also regulated by other gasotransmitters, such as hydrogen sulfide (H_2_S) and carbon monoxide (CO) [40,41,42,43,44,45,46,47,48,49,50,51,52,53,54]. Together with gasotransmitters, vascular endothelium releases prostacyclin, which also represents a key piece in the vasodilation process [55,56,57].

Considering involvement of the NO/cGMP/KATP pathway in the antinociceptive effect of affinin, we hypothesized that this compound might exert a vasodilator effect via activation of gasotransmitters and prostacyclin signaling pathways. Therefore, the aim of this study was to investigate whether affinin, isolated from *H. longipes* roots, was capable of inducing vasodilation and to explore its mechanism of action.

## 2. Results

### 2.1. Phytochemical Study of the Dichloromethane Extract Obtained from H. longipes Roots and Isolation of Affinin

**Figure 1:**
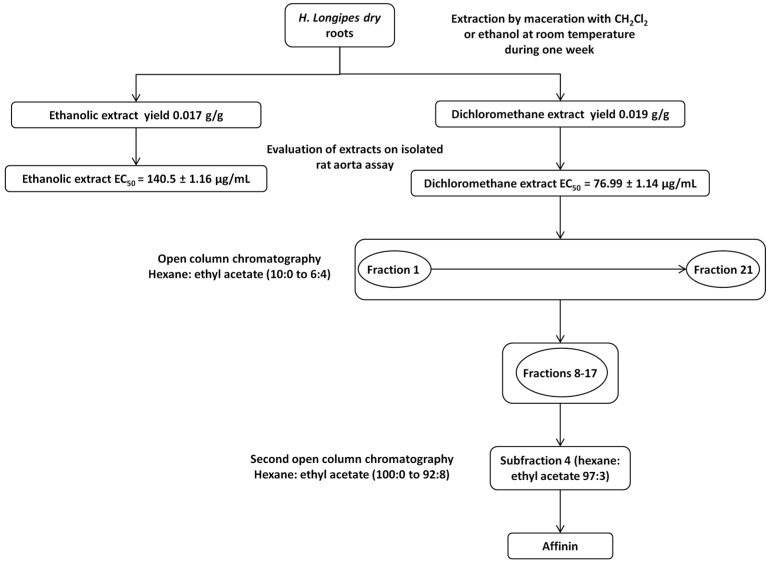
Diagram of the isolation of affinin from the dichloromethane extract of *H. longipes* roots.

Dichloromethane provided a higher yield of extract (19 g/kg roots dry weight) compared to ethanol (17 g/kg roots dry weight). Considering vasodilator potency, the dichloromethane extract was chosen to isolate the bioactive compounds. This extract (100 g) was fractionated by open column chromatography to obtain 21 fractions. Subsequent chromatography of fractions 8–17 resulted in the isolation of 28.5 g of pure affinin (Figure 1).

**Figure 2:**
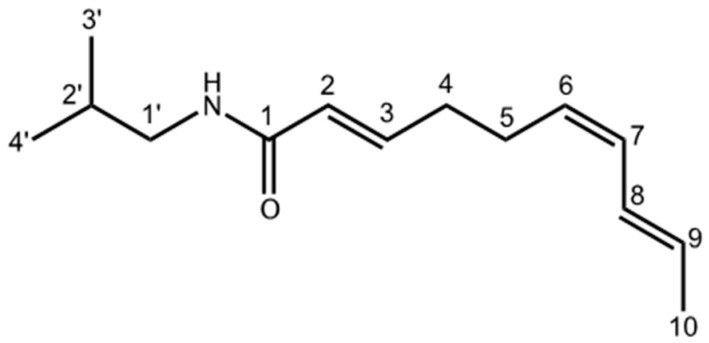
Chemical structure of affinin, the major alkamide in *H. longipes* roots.

Affinin (Figure 2) was identified by comparison with an authentic sample and by comparing its spectroscopic data (^1^H-NMR and ^13^C-NMR) with those previously reported in the literature (Table 1). High performance liquid chromatography/photodiode array detector (HPLC-PDA) analysis of affinin revealed a purity >94%.

### 2.2. Determination of the Vasodilator Effect of H. longipes Extracts and Affinin, and Elucidation of the Mechanism of Action of Affinin

#### 2.2.1. Vasodilator Effect of *H. longipes* Roots Extracts and Affinin

**Figure 3:**
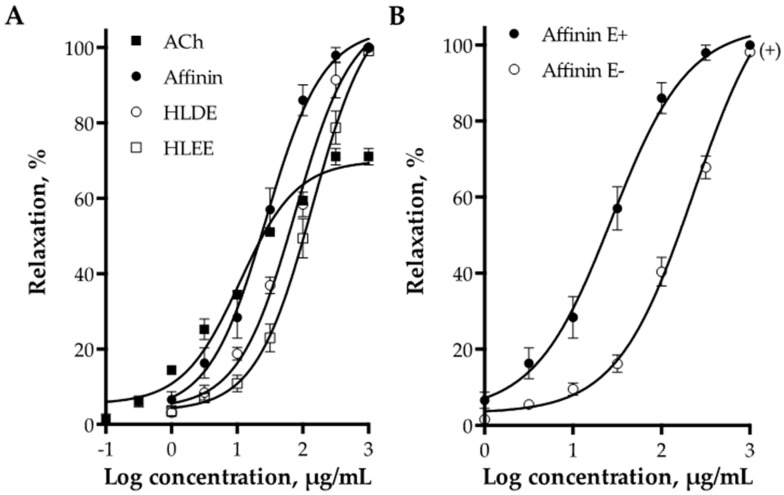
(**A**) Vasodilator effect of the dichloromethane extract (HLDE), the ethanolic extract (HLEE), and affinin from *Heliopsis longipes* roots on intact aortic rings. Acetylcholine (ACh) was used as positive control; (**B**) Concentration-response curves of the vasodilator effect of affinin in the presence (E+) and absence (E−) of endothelium. Values are expressed as mean ± standard error of the mean (SEM) (*n* = 6); + *p* < 0.01.

The dichloromethane and ethanolic extracts of *H. longipes* roots, and affinin, induced a concentration-dependent relaxation of aortic rings with functional endothelium. Figure 3A shows the concentration-response curves for both extracts, affinin, and acetylcholine (ACh), which was used as a positive control. The dichloromethane extract (*E*_max_ = 100% ± 3.11% and EC_50_ = 76.99 ± 1.14 µg/mL) was approximately two fold more potent than the ethanolic extract (*E*_max_ = 100% ± 4.54% and EC_50_ = 140.5 ± 1.16 µg/mL), whereas affinin was significantly more potent than both extracts (*E*_max_ = 100% ± 3.10% and EC_50_ = 27.38 ± 1.20 µg/mL). Affinin turned out to be approximately twenty-five fold less potent than acetylcholine (*E*_max_ = 70.02% ± 1.43% and EC_50_ = 1.094 ± 1.14 µg/mL), however, this alkamide elicited a maximum vasodilator effect greater than that of the positive control (Table 2). Carboxymethylcellulose 1% (CMC), employed as a vehicle, did not show any significant vasodilator effect.

#### 2.2.2. Role of Vascular Endothelium in the Vasodilation Induced by Affinin

Endothelial denudation caused a significant rightward shift in the concentration-response curve of affinin, without affecting the maximal response (*E*_max_ = 100% ± 4.5% and EC_50_ = 231.2 ± 1.13 µg/mL, *p* < 0.01) (Figure 3B).

#### 2.2.3. Involvement of Gasotransmitters in the Vasodilation Produced by Affinin

**Figure 4:**
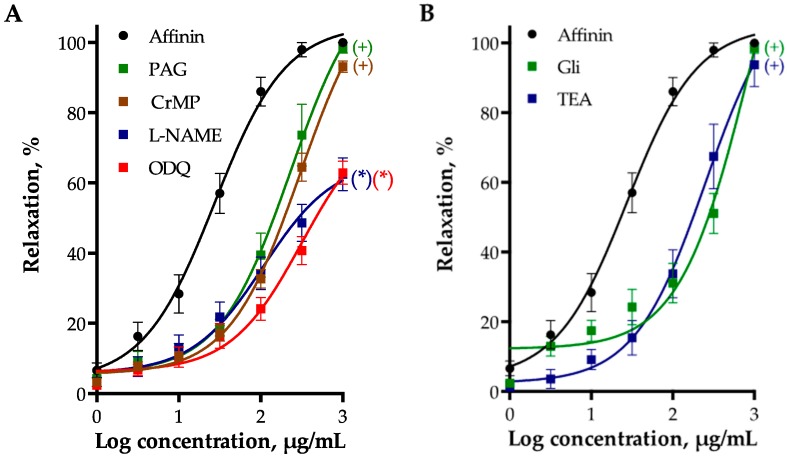
(**A**) Vasodilator effect of affinin in the absence (control) and presence of PAG (1 mM), chromium mesoporphyrin (CrMP, 15 µM), N^G^-nitro-l-arginine methyl ester (l-NAME, 100 µM), and 1*H*-[1,2,4]oxadiazolo[4,3-*a*]quinoxalin-1-one (ODQ, 10 µM) in rat aortic rings; (**B**) Vasodilator effect of affinin in the absence (control) and presence of glibenclamide (Gli, 10 µM) and tetraethyl ammonium (TEA, 1 mM) in rat aortic rings. Values are expressed as mean ± SEM (*n* = 6); + *p* < 0.01; * *p* < 0.001.

The vasorelaxant effect of affinin was significantly reduced by inhibiting endothelial NO synthase (eNOS) with N^G^-nitro-l-arginine methyl ester (l-NAME, 100 µM), heme-oxygenase (HO) with chromium mesoporphyrin IX (CrMP, 15 µM), and cystathionine-γ-lyase (CSE) with dl-propargylglycine (PAG, 1 mM), which indicated that the NO/cGMP, the CO/cGMP, and the H_2_S/K_ATP_ pathways are involved in this effect (Figure 4A). The vasodilator effect of affinin was also significantly reduced by 1*H*-[1,2,4]oxadiazolo[4,3-*a*]quinoxalin-1-one (ODQ, 10 µM), an inhibitor of soluble guanylate cyclase (sGC).

#### 2.2.4. Involvement of K^+^ Channels in Affinin-Evoked Vasodilation

To determine whether activation of K^+^ channels participated in the vasodilatory effect of affinin, the effects of glibenclamide (Gli, a specific blocker of the K_ATP_ channels) and tetraethyl ammonium (TEA, a non-selective K^+^ channel inhibitor) were assessed. Both blockers significantly shifted to the right the concentration-response curve of the vasodilator effect of affinin (Figure 4B), indicating that these channels are involved in its effect.

#### 2.2.5. Effect of PGI_2_/cAMP Pathway on Affinin-Induced Dilation of Rat Aorta

**Figure 5:**
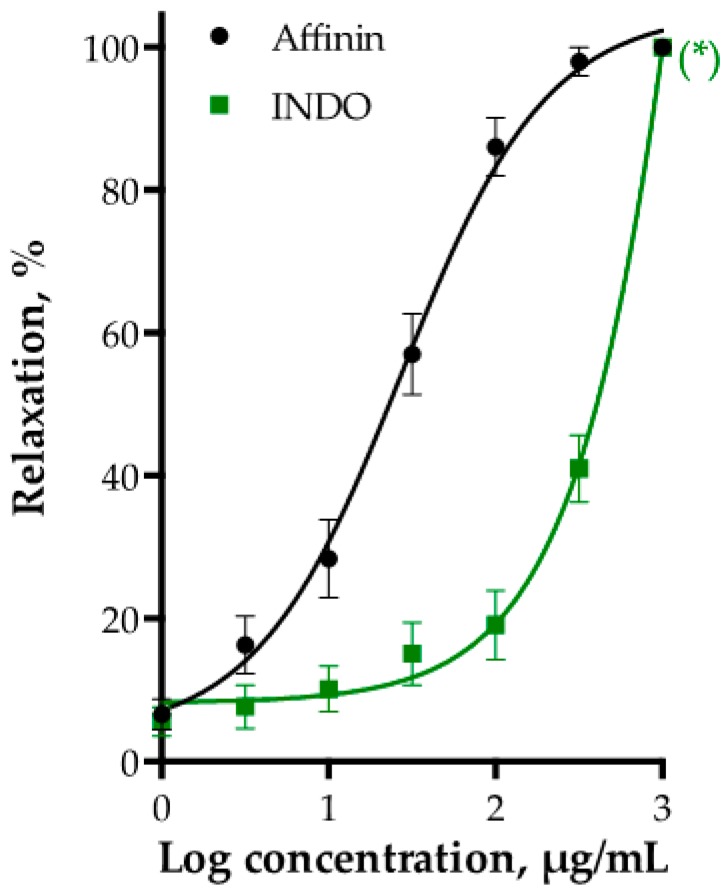
Vasodilatory effect of affinin in the absence (control) and presence of indomethacin (INDO, 10 µM) in rat aortic rings. Values are expressed as mean ± SEM (*n* = 6); * *p* < 0.001.

To test whether the PGI_2_/cAMP pathway was implicated in affinin-induced relaxation, indomethacin (INDO, 10 µM) was used to inhibit cyclooxygenase (COX). INDO pre-treatment significantly reduced the affinin-vasorelaxant effect (Figure 5).

## 3. Discussion

*H. longipes* roots have a long tradition of culinary and medicinal use in Mexico. A number of studies have evidenced that organic extracts obtained from *H. longipes* roots and affinin, their major component, possess interesting biological and pharmacological activities [4,5,6,8,9,16,25,26,28,29,30,31,58]. However, currently, no investigation has been directed toward examining the effect of *H. longipes* root extracts and affinin on the vascular tone.

In the present study, both the dichloromethane and ethanolic extracts from *H. longipes* roots were found to significantly relax the isolated rat aorta. To our knowledge, this has not been previously reported. In 2008, Wongsawatkul et al. [59] described the vasorelaxant effect of four organic extracts (hexane, chloroform, ethyl acetate, and methanol extracts) prepared from aerial parts of *Spilanthes acmella* (Synonym: *Acmella oleracea*) on rat aorta rings. In that study, the ethyl acetate extract exhibited the most potent vasorelaxant effect and, according to the authors, such an effect could be attributed to the presence of polar phenolic and triterpenoid ester compounds. Additionally, the chloroform extract showed the highest maximum vasodilator response. The authors ascribed such effect to triterpenoids and fatty alcohols or esters present in the chloroformic extract. Furthermore, vasodilation induced by the *S. acmella* extracts was completely abolished in the absence of endothelium and significantly reduced in the presence of l-NAME (1 µM) and indomethacin (1 µM), which strongly suggested the participation of the NO and the PGI_2_ pathways [59].

Other studies were carried out to test the effect of oral administration of the *S. acmella* ethanolic flower extract at doses ranging from 50 to 150 mg/kg on sexual performance in male rats. The extract was administered during 28 days and no toxic effects were observed. One of the main findings was the dose-dependent erectile function improvement induced by the extract and its capacity to produce long-term effects, even by day 15 after cessation of the treatment. In the same study, a good correlation was found between these results and a raise on NO levels determined in DS-1 cells (a human corpus cavernosum cell line) cultures stimulated with the *S. acmella* ethanolic extract (100 µg/mL). The authors suggested a possible contribution of affinin and other alkamides present in the extract on the observed effect of improved sexual function [22]. It is a well-known fact that erectile function is mediated by a complex integration of signals, where NO, synthesized by endothelial, inducible, and neuronal NOS, is the most important factor that contributes to vasodilation of the erectile vasculature of the penis [60,61,62].

Regarding our research, based on the vasodilator potency, we selected the dichloromethane extract of *H. longipes* roots to carry out a phytochemical study in order to isolate the bioactive constituents. Chromatographic analysis of the extract led to the isolation of affinin as the major component. This result is consistent with previous studies that have demonstrated that affinin is the main alkamide found in *H. longipes* roots [4,6,8,11,16,25,26]. Of great interest was the finding that affinin elicited a significant vasodilator effect, which was approximately three fold more potent than that of the crude extract. This finding represents the first demonstration that affinin is capable of relaxing the arterial smooth muscle. Considering that affinin was the most abundant constituent of the *H. longipes* dichloromethane extract, it can be inferred that this compound is responsible for the vasodilation induced by the crude extract.

Removal of endothelium significantly decreased, but did not completely block, the vasorelaxation induced by affinin, indicating that both, endothelial-dependent and independent vasodilation pathways are involved in its mechanism of action. The vasorelaxing effect was significantly reduced in the presence of NOS, CSE, and HO inhibitors, which evidenced that activation of the NO/cGMP, H_2_S/KATP, and CO/cGMP pathways contribute to affinin-induced vasodilation. The most relevant inhibition was observed when aortas were preincubated with l-NAME (*p* < 0.001), suggesting that activation of the NO/cGMP pathway plays a more prominent role in the effect of this alkamide than that played by the other two gasotransmitters pathways. Moreover, inhibition of sGC by ODQ (*p* < 0.001) significantly reduced the vasodilatory effect of affinin, revealing that it might directly activate sGC, the main receptor of NO [36]. It is important to point out that CO-sensitive sGC isoforms exist in the vascular smooth muscle [46], therefore CO is also considered to be an important activator of this class of enzymes [37]. Activation of sGC on the smooth muscle cells might underlie, at least in part, endothelium-independent vasodilation caused by affinin.

One of the key mechanisms by which NO, CO, and H_2_S, synthesized in endothelial cells, induce vasodilation is activation of potassium channels located in vascular smooth muscle cells. Regarding nitric oxide-cGMP induced vasodilation, it has been shown that cGMP-dependent protein kinase (PKG) phosphorylates calcium-activated potassium channels (K_Ca_) on the smooth muscle cell membrane leading to a decrease in intracellular calcium concentration [38,39]. This same mechanism is involved in the vasorelaxation produced by CO. Moreover, this gasotransmitter is able to directly activate potassium channels, in particular K_Ca_ [51,52]. On the other hand, H_2_S mediates vasorelaxation via direct opening of K_ATP_ channels [47,48,49,50]. In this study, we assessed whether potassium channel blockers impaired vasodilation provoked by affinin. Glibenclamide and TEA significantly decreased affinin-evoked vasodilation, which confirmed the activation of signaling pathways for NO, CO, and H_2_S.

Indomethacin (*p* < 0.001) caused a significant reduction in affinin-induced vasodilation, suggesting that activation of the PGI_2_/cAMP pathway is also involved in the mechanism of vasorelaxation caused by this alkamide. Along with gasotransmitters, PGI_2_ is secreted by endothelial cells and elicits smooth muscle relaxation by stimulating adenylate cyclase, which subsequently increases cAMP levels. This second messenger activates calcium-activated potassium channels (K_Ca_) via PKA-dependent phosphorylation [55,56]. Evidence from some previous studies suggest that cAMP also enhances K_Ca_ activity by “cross-activation” of PKG [38,54].

According to our results, it is evident that affinin does not only act on a specific type of cell receptor in the arteries. Since its vasodilator effect is not completely blocked in the absence of endothelium, it is clear that this compound activates both endothelium dependent and independent pathways. Our results indicated that affinin is able to activate the NO/cGMP, CO/cGMP, H_2_S/KATP, and PGI2/cAMP signaling pathways, and considering that the triggering of these four signaling pathways depends on the activation of molecular targets located on the endothelium layer, it is very likely that this alkamide might be acting on molecular targets, whose activation leads to an increase in Ca^2+^ levels in the endothelial cells. The chemical structure of *N*-alkylamides or alkamides [63], like affinin [13,64,65] resembles that of fatty acid amides [66,67,68,69], and the endogenous cannabinoid *N*-arachidonylethanolamine or anandamide [70]. This molecule produces a potent vasodilator effect through several proposed mechanisms that include activation of TRPV1 channels and G-coupled receptors, such as CB_1_, CB_2_, and endothelial non-CB_1_/non-CB_2_ [71,72,73]. Herradón et al. [72] showed that the vasodilator effect of anandamide in rat aorta is mainly produced by activation of the endothelial non-CB_1_/non-CB_2_ cannabinoid receptor, which in turn activates the NO/cGMP pathway. Therefore, considering the similarity between the chemical structures of anandamide and affinin, it is quite possible that endothelial non-CB_1_/non-CB_2_ or/and TRP channels may be the putative molecular targets for affinin in the endothelial cells.

Concerning endothelium-independent relaxation induced by affinin, our results indicate that this molecule might directly activate sGC. We can speculate that affinin might also directly activate R_PGI_, although this possibility needs to be confirmed.

**Figure 6:**
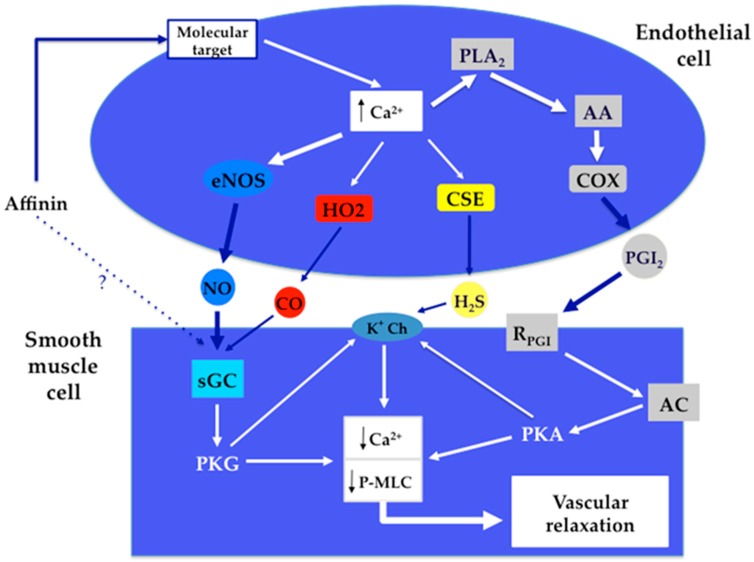
Pathways involved in the vasodilator effect of affinin. PLA_2_, phospholipase A_2_; AA, arachidonic acid; COX, cyclooxygenase; eNOS, endothelial NO synthase; HO2, heme-oxygenase 2; CSE, cystathionine-γ-lyase; sGC, soluble guanylate cyclase; PKG, protein kinase G; AC, adenylate cyclase; PKA, protein kinase A; K^+^ Ch, K^+^ channel; P-MLC, phosphorylated myosin light chain. Black upwards arrow, increased levels; Black downwards arrows, decreased levels. ?, pathway that remains to be confirmed.

Figure 6 shows the proposed signaling pathways involved in the vasodilatory effect of affinin.

The results of the present study have clearly shown that affinin, obtained from *H. longipes* roots, produces vasodilation of rat aorta by activating the NO/cGMP, CO/cGMP, H_2_S/KATP, and PGI_2_/cAMP signaling pathways. This is the first report describing the vasodilator effect of this alkamide and some of the processes involved in its mechanism of action. The median effective concentration to produce vasodilation (EC_50_ = 27.38 µg/mL) falls within the concentration ranges at which this compound elicits other biological and pharmacological activities. Moreover, the EC_50_ obtained for the vasodilator effect of affinin is within the non-cytotoxic concentration range for mammalian cells, however, more cytotoxic studies must be performed in order to establish its possible adverse effects. Besides the other pharmacological properties that have been attributed to affinin, the vasodilator effect is a new interesting activity that might be ascribed to this alkamide. Undoubtedly, these results contribute to support the great therapeutic potential of *Heliopsis longipes* roots and affinin, their main constituent.

## 4. Materials and Methods

### 4.1. Reagents and Chemicals

Reagents and solvents used in the chemical study of *H. longipes* roots were purchased from JT Baker (Phillisburg, NJ, USA). Standards and solvents for the pharmacological assays were obtained from Sigma-Aldrich (St. Louis, MO, USA). CrMP was purchased from Porphyrin Products, Inc. (Logan, UT, USA).

### 4.2. Animals

All experimental protocols were performed in accordance with guidelines of the Mexican Official Standard NOM-062-ZOO-1999 [74], and approved by the Bioethics Committee of the Faculty of Natural Sciences, Autonomous University of Querétaro, México. Wistar male rats (250–300 g) were used for the pharmacological study; they were provided by the Institute of Neurobiology of the National Autonomous University of Mexico, Campus Juriquilla, Querétaro, Qro., Mexico. Animals were housed in standard cages under controlled temperature conditions with a 12:12 h light-dark cycle. Water and food were provided ad libitum.

### 4.3. Plant Material

*H. longipes* (Asteraceae) roots were collected in Peñamiller, Querétaro, Qro., Mexico. The specimens were identified (*H. longipes* vouchers J.E. Castro R.1. and R.2.) and deposited in the Herbario Jerzy Rzedowski (QMEX), Facultad de Ciencias Naturales, Universidad Autónoma de Querétaro, Querétaro, Qro., Mexico.

### 4.4. Preparation of the Extracts Employed for the Pharmacological Evaluation

Air dried *H. longipes* roots were ground to a fine powder. For the preparation of *H. longipes* root extracts, ground plant material (10 g) was subjected to maceration with either dichloromethane or absolute ethanol for one week in a 1:10 ratio (*w*/*v*). This process was repeated three times with fresh solvent. Thereafter, the plant material was filtered and the solvents were removed by rotary evaporation. The extraction yields were: 0.019 g extract/g dried roots for the dichloromethane extract and 0.017 g extract/g dried roots for the ethanolic extract.

### 4.5. Chemical Study of the Dichloromethane Extract Obtained from H. longipes Roots

#### 4.5.1. Fractionation of the Dichloromethane Extract Obtained from *H. longipes* Roots and Purification of Affinin

Dried and ground plant material (7 kg) was extracted with dichloromethane as described above. One hundred grams of the dichloromethane extract were fractionated by column chromatography on normal phase using an open silica gel column (1 kg, Kiesegel 60 Merck 100–230 mesh, 8 × 110 cm). Hexane and ethyl acetate were used as eluents in ratios from 100:0 to 40:60. From this procedure, 472 fractions (250 mL) were collected, monitored by thin layer chromatography (TLC), and grouped into 21 pools according to their chromatographic similarity. TLC analysis of pools 8–17 revealed the presence of a main dark gray spot (*R*f = 0.3, hexane: ethyl acetate 3:2), visualized with an ultraviolet lamp at 254 nm. Spraying TLC plates with a spray solution of anisaldehyde/sulfuric acid (0.5 mL anisaldehyde in 50 mL glacial acetic acid and 1 mL 97% sulfuric acid) developed a bright purple spot, as reported for other olefinic isobutyl-amides [75].

Pools 8–17 were combined (45 g) and further analyzed by open column chromatography (450 g, Kiesegel 60 Merck 100–230 mesh, 4.5 × 120 cm) using a step gradient of hexane and ethyl acetate 100:0 to 90:10. Based on their chromatographic similarity, determined by TLC, fractions eluted with hexane: ethyl acetate 97:3 were combined and evaporated to dryness in vacuo leaving a residue of 28.5 g of an apparently pure compound. The purity of the isolated compound was confirmed by HPLC-PDA, using an HPLC chromatograph (Waters 600 Associates, Milford, MA, USA) coupled to a photodiode array detector (Waters 2998). This analysis was carried out on a XBridge C18 (4.6 × 100 mm 3.5 µm) column. The flow rate of the mobile phase (acetonitrile/water 44:56 *v*/*v*) was 0.5 mL/min with column temperature of 30 °C and detection wavelength of 229 nm.

#### 4.5.2. Determination of the Chemical Structure of Affinin

Chemical structure of the purified compound was elucidated by analysis of its proton nuclear magnetic resonance (^1^H-NMR) and carbon-13 (^13^C-NMR) spectra (Table 1). Nuclear magnetic resonance (NMR) spectra were taken on a Varian VNMRS 400 spectrometer with tetramethylsilane (TMS) as internal standard. Affinin was identified by comparing its spectroscopic constants with those reported in the literature [20,21].

### 4.6. Determination of the Vasodilator Effect and Elucidation of the Mechanism of Action of Affinin

#### 4.6.1. Isolated Rat Aorta Assay

The rats were killed by decapitation. The thoracic aorta was surgically removed and placed in a Petri dish containing ice-cold (4 °C) Krebs-Henseleit solution with the following composition (mM): 126.8 NaCl; 5.9 KCl; 1.2 KH_2_PO_4_; 1.2 MgSO_4_; 5.0 d-glucose; 30 NaHCO_3_; 2.5 CaCl_2_ (pH 7.4), bubbled with a mixture of carbogen (95% O_2_ and 5% CO_2_). Then, the intraluminal space of aorta was rinsed with fresh solution to prevent clot formation, cleaned from surrounding connective tissue, and sliced into rings (3–4 mm in length). Aortic rings were mounted between two metallic hooks, with one being fixed and the other attached to an isometric transducer, and placed into organ baths chambers containing pre-warmed Krebs-Henseleit solution (37 °C) gassed with carbogen. The aortic segments were allowed to equilibrate for 60 min under a resting tension of 1.5 g. During the resting period, the organ bath solution was exchanged every 10 min. In order to stimulate the vascular smooth muscle, the tissues were contracted with KCl solution (100 mM). Once a stable contractile tone was reached, the bathing medium was replaced every 10 min to restore the initial resting tension of 1.5 g. Afterwards, the aortic rings were contracted with 1 µM l-phenylephrine (Phe); the contractile force induced was defined as 100%, and once the plateau was reached, the test substances were cumulatively added. Acetylcholine (ACh), dissolved in distilled water, was evaluated in a concentration range of 0.2 ng/mL–2 mg/mL; while affinin and the extracts, dissolved in vehicle (carboxymethylcellulose 1% in distilled water), were tested in a concentration range of 1 µg/mL–1 mg/mL. When used, pharmacological inhibitors were added to the organ bath chambers 20 min before the addition of Phe. The changes in tension caused by the tested concentrations were detected by Grass FT03 force transducers coupled to a Grass 7D Polygraph; they were expressed as percentages of relaxation based on the contraction generated by adding Phe [76].

#### 4.6.2. Participation of the Endothelium in the Vasodilator Response of Affinin

To determine whether the vasodilator response of affinin was dependent on the vascular endothelium, assays on aorta segments without endothelium were performed. In these experiments the endothelial layer was removed by flushing the lumen of aorta with 0.2% desoxycholate in saline solution 0.9%, as reported previously [76]. The absence of endothelium was confirmed at the start of the experiment, showing that the addition of 1 µM of acetylcholine (ACh) did not induce more than 5% relaxation. Once the cumulative concentrations of affinin were added to the bath chambers, as described above, sodium nitroprusside (100 µM) was added to the chambers to demonstrate that the artery was still capable of relaxation.

#### 4.6.3. Evaluation of the Participation of the Gasotransmitters and Prostacyclin Signaling Pathways in the Vasodilator Response of Affinin

Involvement of the main gasotransmitters pathways in the vasodilator effect evoked by affinin was assessed by incubating intact endothelium aortic rings for 20 min in the presence of inhibitors of specific enzymes of each of these pathways: (1) NO/cGMP pathway: 100 µM N^G^-nitro-l-arginine methyl ester (l-NAME, inhibitor of eNOS) or 10 µM 1*H*-[1,2,4]oxadiazolo[4,3-*a*]quinoxalin-1-one (ODQ, inhibitor of sGC); (2) H_2_S/K_ATP_ channel pathway: 1 mM dl-propargylglycine (PAG, inhibitor of CSE); and (3) HO/CO pathway: 15 µM chromium mesoporphyrin IX (CrMP, inhibitor of HO) [40,41,42,43,44,49,52,77].

To determine the involvement of the prostacyclin pathway in the vasodilator effect of affinin, aortic segments were pre-incubated for 20 min in the presence of 1 µM indomethacin (INDO, inhibitor of COX) [78,79]. In addition, to assess whether activation of K^+^ channels was involved in the vasodilation produced by affinin, the effect of pretreatment with the non-selective potassium channel blocker, 1 mM tetraethyl ammonium (TEA) and 10 µM glibenclamide (a specific blocker of the K_ATP_ channels) was evaluated [80,81].

### 4.7. Statistical Analysis

Evaluations of each concentration of the tested substances were performed on aortas obtained from at least three different rats (*n* = 6). All values are expressed as the mean ± standard error of the mean (SEM). The resulting data obtained from each evaluation were fitted to a sigmoidal equation, plotted, and analyzed to calculate EC_50_ (GraphPad Prism 7.02, San Diego, CA, USA). These results were subjected to one-way analysis of variance (ANOVA) using the statistical program GraphPad Prism 7.02, followed by the Tukey test to evaluate any significant differences between the means. Values of + *p* < 0.01 or * *p* < 0.001 were considered to be significant.

## 5. Conclusions

Our study provides a heretofore unknown evidence that affinin, isolated from *H. longipes* roots, is capable of inducing vasodilation via mechanisms that involve activation of gasotransmitters and prostacyclin signaling pathways. The NO/cGMP and PGI_2_/cAMP pathways appear to play a more prominent role than either the H_2_S/KATP pathway or the CO/cGMP pathway in affinin-evoked vasorelaxation. Undoubtedly, this molecule deserves further investigation in order to completely understand its mechanism of action. The results derived from this study suggest that affinin is a promising molecule for the development of drugs useful in the prevention and/or treatment of cardiovascular diseases, particularly when considering that it has an adequate lipophilicity that allows it to permeate skin and oral mucosa, and reach blood circulation.

## Figures and Tables

**Table 1:** ^13^C-NMR (400 MHz) and ^1^H-NMR (400 MHz) spectral data of affinin.

H	δ_ppm_	C
1	-	166.15
2	5.80 (1H, br d, *J* = 16.0, 8.0 Hz)	124.30
3	6.80 (1H, dt, *J* = 16.0, 8.0 Hz)	143.51
4	2.28 (4H, m)	32.20
5	2.28 (4H, m)	26.49
6	5.25 (1H, dt, *J* = 10.7, 7.1 Hz)	127.73
7	5.94 (1H, dd, *J* = 12.0 Hz)	129.52
8	6.25 (1H, br dd, *J* = 16.0, 4.0 Hz)	126.79
9	5.67 (1H, dq, *J* = 16.0, 6.0 Hz)	130.00
10	1.76 (3H, d, *J* = 6.0 Hz)	18.39
NH	5.47 (br s)	-
1′	3.13 (2H, dd, *J* = 6.0, 6.0 Hz)	46.97
2′	1.80 (1H, m)	28.68
3′	0.93 (6H, d, *J* = 6.7 Hz)	20.23
4′	0.93 (6H, d, *J* = 6.7 Hz)	18.40

Affinin was recorded in CDCl_3_. Integrations, multiplicity, and coupling constants of protons are shown in parentheses.

**Table 2:** Vasodilator effect of *Heliopsis longipes* roots extracts and affinin on rat aorta.

Compound	*E*_max_ (%)	EC_50_ (µg/mL)
Dichloromethane extract	100 ± 3.11	76.99 ± 1.14
Ethanolic extract	100 ± 4.54	140.5 ± 1.16
Affinin	100 ± 3.10	27.38 ± 1.20
ACh	70.02 ± 1.43	1.094 ± 1.14

Data are expressed as mean ± SEM (*n* = 6). Acetylcholine (ACh) is presented as positive control.
